# Risk prediction of developing venous thrombosis in combined oral contraceptive users

**DOI:** 10.1371/journal.pone.0182041

**Published:** 2017-07-27

**Authors:** Aaron McDaid, Emmanuelle Logette, Valérie Buchillier, Maude Muriset, Pierre Suchon, Thierry Daniel Pache, Goranka Tanackovic, Zoltán Kutalik, Joëlle Michaud

**Affiliations:** 1 Institute of Social and Preventive Medicine, University Hospital of Lausanne, Lausanne, Switzerland; 2 Swiss Institute of Bioinformatics (SIB), Lausanne, Switzerland; 3 Gene Predictis SA, EPFL Innovation Park, 1015 Lausanne, Switzerland; 4 AIx Marseille Univ, INSERM, INRA, NORT, Marseille, France; 5 APHM, Hôpital de la Timone, Service d'hématologie biologique, Marseille, France; Institut d'Investigacions Biomediques de Barcelona, SPAIN

## Abstract

**Background:**

Venous thromboembolism (VTE) is a complex multifactorial disease influenced by genetic and environmental risk factors. An example for the latter is the regular use of combined oral contraceptives (CC), which increases the risk to develop VTE by 3 to 7 fold, depending on estrogen dosage and the type of progestin present in the pill. One out of 1'000 women using CC develops thrombosis, often with life-long consequences; a risk assessment is therefore necessary prior to such treatment. Currently known clinical risk factors associated with VTE development in general are routinely checked by medical doctors, however they are far from being sufficient for risk prediction, even when combined with genetic tests for *Factor V Leiden* and *Factor II G20210A* variants. Thus, clinical and notably genetic risk factors specific to the development of thrombosis associated with the use of CC in particular should be identified.

**Methods and findings:**

Step-wise (logistic) model selection was applied to a population of 1622 women using CC, half of whom (794) had developed a thromboembolic event while using contraceptives. 46 polymorphisms and clinical parameters were tested in the model selection and a specific combination of 4 clinical risk factors and 9 polymorphisms were identified. Among the 9 polymorphisms, there are two novel genetic polymorphisms (rs1799853 and rs4379368) that had not been previously associated with the development of thromboembolic event. This new prediction model outperforms (AUC 0.71, 95% CI 0.69–0.74) previously published models for general thromboembolic events in a cross-validation setting. Further validation in independent populations should be envisaged.

**Conclusion:**

We identified two new genetic variants associated to VTE development, as well as a robust prediction model to assess the risk of thrombosis for women using combined oral contraceptives. This model outperforms current medical practice as well as previously published models and is the first model specific to CC use.

## Introduction

Venous thromboembolism (VTE), which includes deep vein thrombosis (DVT) and pulmonary embolism (PE), occurs in 1–2 per 1'000 individuals per year. The incidence increases with age, from 1 in 100'000 in children to 1 in 10'000 individuals in the reproductive age, 1 in 1'000 individuals at the age 50 to 60 and 1 in 100 over 75 years old [1]. VTE is a complex multifactorial disease influenced by several acquired or inherited conditions. The acquired conditions include a large number of risk factors such as surgery and trauma, prolonged immobilization, cancer, myeloproliferative disorders, and even pregnancy and post-partum [2]. Weight, age, smoking status and hormonal treatment are all additional environmental factors associated with an increased risk of VTE.

The inherited conditions include mutations in the diverse well-known clotting anticoagulant or thrombolytic factors genes, such as the *Factor V Leiden* (*F5*) gene, and the *prothrombin Factor II* (*F2*) gene. Such mutations can also be present in genes coding for proteins C and S, however despite the fact that they increase the risk of developing venous thrombosis significantly, they are rare and most of them are practically private [3]. Other likely inherited causes include a possible increase in the expression of procoagulant factors such as factor VIII, von Willebrand factor, and factors IX and XI [4]. In addition, non-O ABO blood groups, with the exception of the A2 group, were demonstrated to increase the risk of developing thrombosis. Many other additional genetic variants, present in the genes *FGG*, *GP6*, *KNG1*, *PROCR*, *SLC44A2*, *STXBP5* and *TSPAN15*, among others, were associated with an increased risk of venous thrombosis [5].

Over 100 million women worldwide use combined estroprogestative contraceptives (CC), due to their very high effectiveness in reducing the risk of unwanted pregnancy and their beneficial effect on diverse symptoms related to women's cycle. Nonetheless, these contraceptives also increase the risk of blood clotting substantially, which can ultimately lead to DVT and PE [6]. Newer generations of CC, the so-called 3rd and 4th generation CC (pills containing norgestimate, gestodene, desogestrel or drospirenone as progestin), are usually better tolerated by women but importantly, they increase the risk of developing VTE even more than the older preparations of the so-called 2^nd^ generation (levonorgestrel containing-pills).

The incidence of thrombosis among CC users is around 1‰ per year [7]. In France alone, where over 3 million women aged 15–49 use CC, the National Agency for the Safety of Drugs and Health Products reports every year over 2'500 cases of DVT, 850 cases of PE, and 20 cases of death linked to contraceptive pills. Taking into account the incidence of thrombosis among contraceptive pill users and number of women using them, it is estimated that 22'000 DVT related to CC occur each year in Europe. Thus, one of the major challenges for healthcare professionals is to identify women at risk of developing blood clotting disease related to CC such as DVT and PE, and advise them on alternative contraception methods.

As the standard of care nowadays, prescribing physicians assess the risk of thrombosis using clinical parameters, mostly focusing on age, body mass index, smoking habits and personal and familial history of DVT or related diseases that are known risk factors for VTE development. However, diverse studies demonstrate that clinical informations, notably familial history, are insufficient to reliably estimate risk of VTE [8, 9]. When the familial history of thrombosis is positive, physicians might use the first-level laboratory test for thrombophilia screening that includes analysis of only 2 genetic risk factors: the *F5-Leiden* and the *F2* mutations; eventually, some laboratories, also include genetic tests allowing to assess for the ABO blood group. Widely-accepted evidence of haemostatic abnormalities associated with thrombophilia includes the following parameters: antithrombin deficiency, protein S deficiency, protein C deficiency, *F5-Leiden* mutation, *F2* mutation, non-O ABO blood group and high levels of factor VIII dysfibrinogenaemia [10]. Though *F5-Leiden* and *F2* mutation are well-established risk factors for thrombosis development, they explain less then one third of the inherited risk to develop thrombosis. Precisely, *F5-Leiden* is present among 20% of patients that develop thrombosis, whereas only 6% of patients carry the *F2* mutation [10]. Therefore, genomic assessement that takes into account other polymorphisms associated with VTE development is mandatory.

## Materials & methods

### Population studied

The population described in this study has been designed to investigate the clinical and genetic factors that affect the risk of VTE in women taking CC. The study includes 794 female cases who have developed at least one episode of VTE while taking CC. These cases are part of the previously described PILl Genetic RIsk Monitoring (PILGRIM) study [11], in which the method used to confirm the occurence of thrombosis is defined. 828 control women were also collected from different sources: 523 are part of the PILGRIM study; 174 are part of the CoLaus study [12], 56 were recruited between 1997 and 1998 in south of France among healthy volunteers and the remaining controls were recruited by established medical clinicians between 2012 and 2016 among Swiss population. The last two groups of controls include any woman of childbearing age who was using CC at the time of collection and did not have a thrombotic event prior to sample collection time. These women were not recruited as part of thrombophilia screening and are unrelated to thrombotic patients. Nonetheless some of them have described a family history at the time of collection (19/128). The PILGRIM study controls presented a selection bias due to having been recruited as part of thrombophilia screening due to family history [11] and several variables (family history, F5 and F2) were not used as such, as described below. All control women are taking CC but have not developed VTE by the time of the genotyping investigation. This study involved human subjects and was carried out in accordance with the tenets of the Declaration of Helsinki; all participants signed an informed consent and data were anonymised. The procedures regarding the collect of PILGRIM samples were reviewed and approved by the Assistance Publique des Hopitaux de Marseille insitutional review committee. The CoLaus study was approved by the Ethics Committee of Lausanne University.

### Genotyping

46 SNPs were selected according to their association with VTE development or hormone metabolism (principaly estrogens) as described in the literature. These 46 SNPs were genotyped using Illumina GoldenGate technology and assessed using Illumina BeadXpress and GenomeStudio V2011.1 software. Clusters for each SNP were curated manually and undetermined samples were further genotyped using Sanger sequencing. SNP rs1053878 was genotyped using RFLP-PCR; in more details, the DNA region was amplified with the following primers (Forward: 5’-GCCACCGTGTCCACTACTATG-3’ and Reverse: 5’- GTCCACGCACACCAGGTAAT-3’) and the amplicons were digested with PvuII restriction enzyme. Controls from the CoLaus cohort were previously genotyped as described [13]. For the CoLaus controls, proxys (*r*^*2*^ > 85%) were used for 9 SNPs (rs4572916 for rs10029715, rs8176704 for rs1053878, rs3736455 for rs13146272, rs6018 for rs1800595, rs4253417 for rs2289252, rs11038993 for rs3136516, rs2169682 for rs7082872, rs687621 for rs8176719 and rs2069952 for rs9574). Genotyping data for rs1799963 was missing in the CoLaus study. Genotpying data for rs6025 and rs1799963 of the 523 control samples from the PILGRIM study were ignored to avoid selection bias due to having been recruited as part of thrombophilia screening due to family history [11]. Allele frequencies of the 46 SNPs in the controls were consistent with the ones observed in the European subsample of the 1000 Genomes panel [14].

### Clinical characteristics

Age and smoking status were determined at the time of VTE for cases and at the time of DNA collection for controls. BMI was dertermined at the time of consultation for both cases and controls. Family history was defined as positive when at least one first-degree relative has suffered VTE. Information on family history for the 523 control women from the PILGRIM study was not used as such because of the recruitment bias [11]. All women included in this study took oral combined contraceptive.

### Statistical analyses

The study population was randomly divided 10’000 times into a training set and a test set of equal size. For every sample split missing values were imputed by a random draw from the non-missing values present in the control samples. Once missing values were imputed, we applied step-wise logistic regression model selection (as long as the Akaike Information Criterion (AIC) was improved) to each training set to select variables and assign coefficients. The fitted model was then applied to the test sets to estimate the predictions of the model in an out-of-sample setting. Across the 10’000 runs the average number of selected variables was 18.1. Two variables were selected over 99.9% of the time (rs6025 and rs1799963). When a run did not select a variable (i.e. we had no evidence that the coefficient is significantly different from zero) its coefficient was set to zero, equivalent to an odds-ratio of 1. The final model coefficients are estimated as the median values of the coefficients across the 10,000 runs. This model consists of 13 variables (including 9 SNPs) with non-zero median values. The corresponding standard error (SE) for each of the 13 coefficients is the median standard error across those runs (out of the 10,000) when the variable was selected into the model. Confidence intervals and p-values were derived from the coefficients and standard errors (SEs) in the standard manner.

We compared our 9-variable genetic prediction model (including only SNPs) to previously published genetic models [8, 15]. For a fair evaluation, in each random data split (and imputation) the coefficients of each model (including our 9-variable genetic model) were estimated in the training set and the predictions were evaluated in the test set based on the Area Under the receiver operator characteristic Curve (AUC). AUC is equal to the probability that the predictor value of a positive test ranks higher than that of a negative test in order to discriminate the women at risk to the women without risk. The AUC ranges from 0.5 (50%—no predictive value) to 1 (100%—perfect discrimination) [16].The final AUC for each model is its median AUC across the 10,000 random data splits.

## Results

The clinical characteristics of the population of women taking CC described here are defined in Table 1. Age distribution is similar between cases and controls as demonstrated by Wilcoxon rank-sum test (p-value = 0.1). Five parameters are statistically different between both populations, including 3 clinical variables (BMI, family history and smoking status) and two thrombophilia markers, FV-Leiden and prothrombin (F2). Although we cannot demonstrate that these differences are not due to selection bias, all five characteristics are known risk factors for VTE development, thus the minor observed differences are not surprising. The modest differences observed in our samples reinforce the current evidence that clinical information is not sufficient to distinguish women at risk to develop VTE [8, 9].

**Table 1:** Clinical characteristics of the population.

	Cases (n)	%/SD	Controls (n)	%/SD	p-value^6^
**Total number**	794	49%	828^1^	51%	
**VTE**	794				
**DVT**	600	75.5%			
**PE**	194	24.5%			
**Age (mean)**	32 [17–49] ^+^	SD: ± 9.6^+^	31.5 [18–51] ^+^	SD: ± 9.0^+^	0.1
**BMI (mean)**	24 [18–37] ^+^	SD: ± 5.2^+^	23 [17.5–33.5] ^+^	SD: ± 4.2^+^	**6.6E-06**
**Family history of VTE**	222	28%	19(317)^2^	15(38)^2^%	**1.7E-03**
**Smoking**	260	33%	206	25%	**4.7E-04**
**Cancer**	6	0.7%	2^3^	0.2%	0.4
**Autoimmune disease**	8	1%	4^3^	0.7%	0.65
**Thrombophilia factors:**					
**Protein C**	20	2.5%	7^3^	1.6%	0.1
**Protein S**	10	1.2%	12^3^	0%	0.15
**Antithrombin**	6	0.8%	2^1^	0.5%	0.4
**F5-Leiden**	132	16.5%	10(98)^4^	3(13)^4^%	**3.3E-09**
**Prothrombin (F2)**	80	10%	3(64)^5^	2(8)^4^%	**3.8E-03**

^+^ 95%CI (in brackets) and Standard deviation (SD) are indicated for these parameters

^1^ The total number of controls differs depending on the variable as indicated in ^2^ and ^3^.

^2^ This variable was set as missing or was missing for 700 control women as indicated in M&M and the total number of controls used here is 128 controls. The number indicated in brackets is the original number before correction for bias.

^3^ This parameter is missing for 305 control women.

^4^ This variable was set as missing for 523 control women as indicated in M&M and the total number of controls used here is 305 controls. The number indicated in brackets is the original number before correction for bias.

^5^ This variable was set as missing for 697 control women as indicated in M&M and the total number of controls used here is 131 controls. The number indicated in brackets is the original number before correction for bias.

^6^ p-values calculated using Wilcoxon rank-sum test to compare cases to controls.

46 SNPs selected according to their association with VTE development or hormone metabolism (principaly estrogens), as described in the literature, were successfully genotyped in the 1622 women involved in this study. Familial history and genotyping data of rs6025 and rs1799963 of 523 control women were treated as missing in order to avoid an ascertainment bias. To make sure that the frequency of each SNP in our control population corresponds to the frequencies expected in a general Caucasian population, we compared the allele frequencies (AF) observed in our control population to the ones reported in 1000 genomes project [14]. The frequencies were similar (S1 Table, Fisher p-values > 0.01) for all but two SNPs (rs1593812 and rs429358, which were discarded from further analysis) suggesting that our control population reflects a general Caucasian population and that the genotyping is of high quality.

Logistic regression models were fitted step-wise to find the optimal (in terms of AIC) multivariate model in the 10,000 training sets. By averaging these 10,000 models, we identified 4 clinical variables as risk factors contributing to the prediction of the risk of VTE in our population. Age, BMI, smoking status and family history were selected and had significant p-values (Table 2, p-values < 0.05). 9 out of the 44 tested SNPs were in the averaged model and also significantly associated with the development of thrombosis (Table 2). The reported p-values survive 5% false discovery rate (FDR) control. Among these nine SNPs, as expected, *F5-Leiden* (OR = 6.46, CI = [3.46–8.37]) and *F2* (OR = 5.32, CI = [2.66–7.9]) mutations are long-known risk VTE factors. Further five SNPs including rs2289252 (*F11*), rs710446 (*KNG1*), rs9574 (*PROCR*) and rs8176719/rs8176750 (tagging ABO subtypes) have been recently associated with VTE (Table 2) [17–20]. The final two of the nine polymorphisms, rs1799853 (*CYP2C9*) and rs4379368 (*SUGCT*), have not been described before to impact VTE development. No interactions among selected parameters were identified to be significant.

**Table 2:** Clinical and genetic parameters selected in the Pill Protect® model.

Variable	Gene (when applicable)	Effect allele (when applicable)	Mean (frequency) among cases	Mean (frequency) among controls	OR	95% CI	p-value
**Family history of VTE**			28^1^	15^1^	2.13	1.61–2.83	1.4E-07
**Smoking**			33^1^	25^1^	1.63	1.27–2.09	1.3E-04
**BMI**			24^2^	23^2^	1.07	1.04–1.09	3.2E-07
**Age**			32^2^	31.5^2^	1.01	1.001–1.03	0.03
**rs6025**	*F5*	A	0.09	0.02	6.46	4.04–10.3	5.8E-15
**rs1799963**	*F2*	A	0.05	0.01	5.32	3.01–9.31	7.39E-09
**rs8176719**	*ABO*	I	0.50	0.41	1.52	1.28–1.80	1.71E-06
**rs2289252**	*F11*	T	0.49	0.42	1.34	1.14–1.58	3.5E-04
**rs1799853**	*CYP2C9*	T	0.15	0.12	1.54	1.21–1.94	3.5E-04
**rs9574**	*PROCR*	G	0.57	0.51	1.25	1.07–1.47	0.0052
**rs8176750**	*ABO*	D	0.05	0.07	0.60	0.42–0.85	0.0043
**rs4379368**	*SUGCT*	T	0.11	0.08	1.35	1.03–1.80	0.032
**rs710446**	*KNG1*	G	0.46	0.43	1.22	1.04–1.43	0.016

^1^ Percentage of cases and controls with the corresponding clinical factor

^2^ Mean of the corresponding clinical factor across the cases or controls

**Fig 1:**
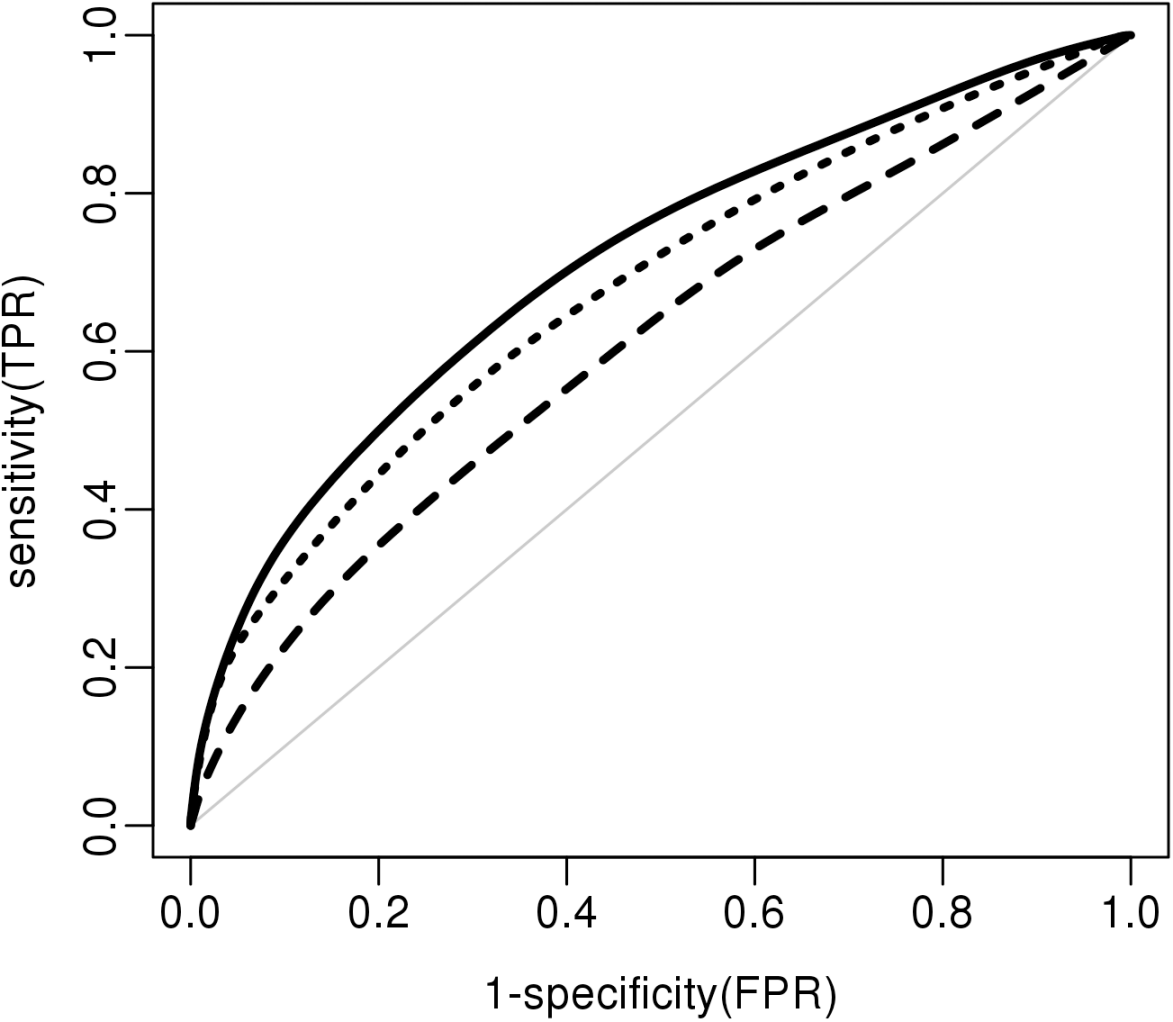
ROC (AUC) curves for Pill Protect models. The clinical (dashed line), genetic (dotted line) and combined models (black line) are indicated. The light grey line represents the reference line (AUC 0.5).

We estimated the out-of-sample performance of the set of 13 combined parameters as well as the clinical- and genetic-only models separately (Pill Protect® models). The ROC curves for the clinical, genetic and combined models are represented in Fig 1. The clinical model gives an AUC of 0.61 (0.58–0.64) and the genetic variables alone give an AUC of 0.68 (0.65–0.71). Combining both clinical and genetic parameters increase the AUC to 0.71 (0.69–0.74). To compare these results with the current best practice, based on an oral anamnesis of the patient, we estimated coefficients for the clinical variables from a meta-analysis of the literature using weighted means (S2 Table, MD algorithm). In some cases, medical doctors may request a thrombophilia status that includes the genotyping information for *F5-Leiden* and *F2* mutations (rs6025 and rs1799963). We, therefore, also compared our model to a model that contains the previous clinical variables and coefficients for these two SNPs obtained from the literature (S2 Table, MD-gen algorithm). The MD model reached an AUC of 0.61 in our studied population that is similar to our clinical-only model. After adding genetic information to the MD model, the MD-gen model reaches an AUC of 0.67 in our studied population. Our combined model achieved significantly higher performance than any model we could derive from the literature (Table 3).

**Table 3:** Out-of sample AUC values for various published- and our models applied to our studied population.

Model	AUC	95% CI
Pill Protect® clinical model	0.61	0.58–0.64
Pill Protect® genetic model	0.68	0.65–0.70
Pill Protect® combined model	0.71	0.69–0.74
MD model	0.61	0.60–0.62
MD-gen model	0.67	0.66–0.68
Bruzelius genetics	0.65	0.63–0.68
De Haan genetics	0.64	0.62–0.68

**Fig 2:**
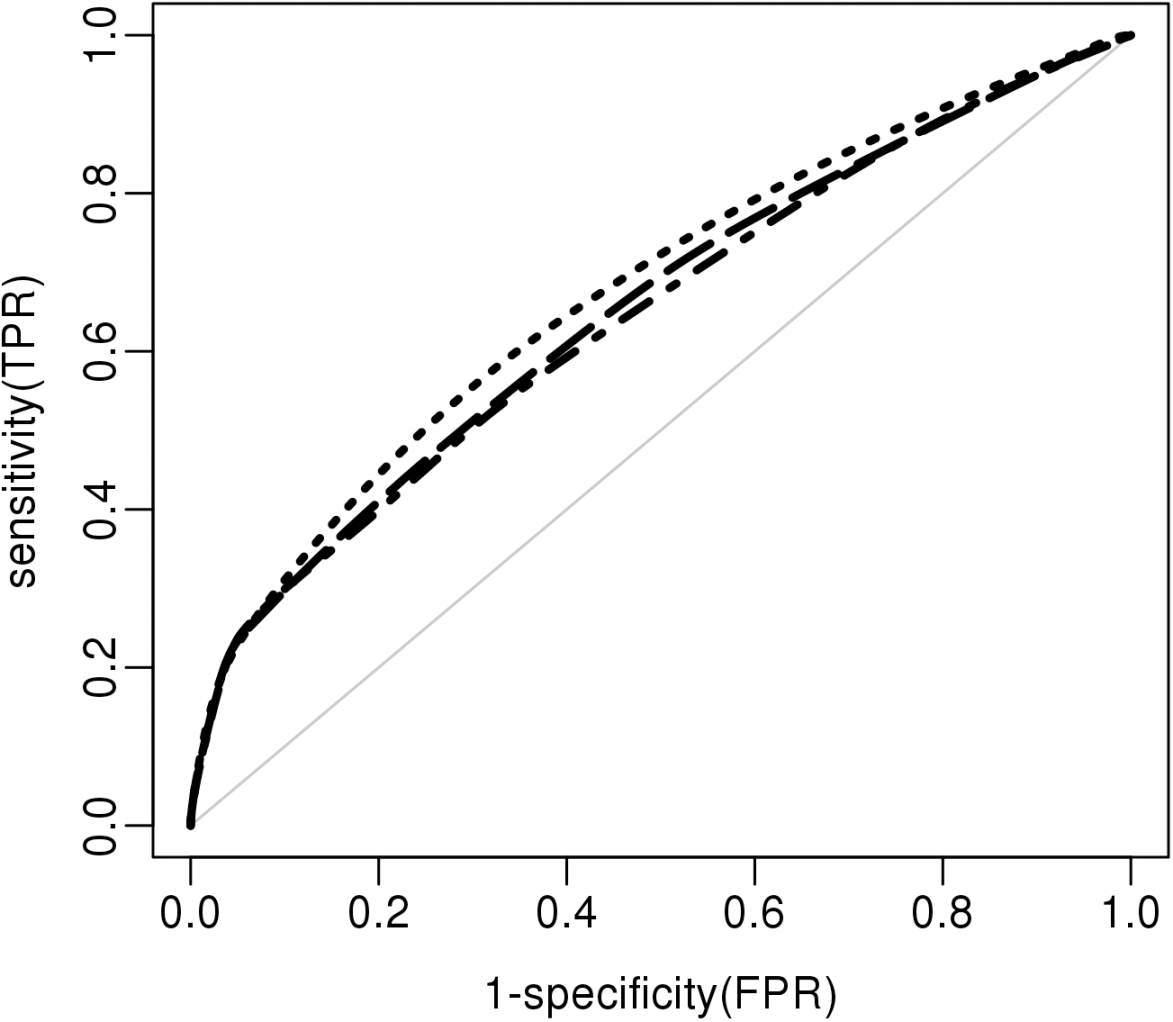
ROC (AUC) curves for the Pill Protect® and published genetic models. The models described in De Haan et al. (dot-dashed line), in Bruzelius et al. (long dashes) and in this paper (dotted line) are indicated. The light grey line represents the reference line (AUC 0.5).

Previous studies have modelled VTE risk using different combinations of parameters although none are specific to the use of CC. Because the whole set of clinical parameters used by the other models was not available, we compared only the genetic models. The genetic score described by De Haan et al. [8] is based on 5 SNPs (rs6025, rs1799963, rs8176719, rs2066865, rs2036914). All of these SNPs are present in our current study, although only 3 of them are used in our final model. Applying this 5-SNP model to our population yielded an AUC of 0.64 (0.62–0.68) (Table 3 and Fig 2), which is less than the described AUC on MEGA and LETS cohorts (0.69 and 0.67 respectively) due to winners curse. The genetic score described by Bruzelius et al. [15] is based on 7 SNPs (rs6025, rs1799963, rs514659, rs2289252, rs1799810, rs710446, rs2066865) and 4 interactions. Among the 7 SNPs, 4 are present and one (rs514659) has a good proxy (rs8176719) in our Pill Protect® model, and one (rs2066865) is not part of our model but is present in our dataset. The last SNP (rs1799810) is absent from our dataset and was, therefore, not used in the comparison. However, given the small (and least significant) coefficient reported by Bruzelius, it would probably not affect significantly the performance. This is confirmed by the fact that genetic score associated with this set of six SNPs reaches an AUC of 0.65 (0.63–0.68) in our study (Table 3 and Fig 2), which is very similar to what was described by Bruzelius et al. (0.66; [0.64–0.68]). Still both AUC values are significantly below the 0.68 AUC of our 9-SNP genetic model.

## Discussion

In this study, we determined a new combination of parameters that predicts the risk of VTE in women using CC. This combination outperformed significantly previously published models as well as the clinical evaluation currently used by medical doctors. We also identified two new genetic markers associated with the development of VTE in our population.

The risk of VTE development upon CC use is presently assessed by an oral anamnesis and based on physician’s experience. In the presence of clinical risk factors and/or family history, some medical doctors may request a thrombophilia status that tests for the well-established markers FV-Leiden and Prothrombin. The current incidence of 1‰ of VTE per year in CC users indicates that the risk assessement needs to be improved. Analysing genetic and clinical data for a population of women using CC, we were able to calculate a risk score that outperfoms a model that simulates the current empirical approach even when combined with additional information on FV-Leiden and Prothrombin. Our predictor Pill Protect®, including 9 genetic markers in combination with 4 clinical factors, was able to reach an out-of-sample AUC of 0.71. The use of these 9 genetic markers also outperformed the combination of markers previously published by others [8, 15]. A thrombophilia status would be complementary to the risk score approach described here, as a functional test in the presence of clinical suscpiscion, in order to take into account rare mutations such as the one present in *Protein S*, *Protein C* or *Antithrombin* genes.

Our study presents some limitations regarding the identification of rare polymorphisms and rare mutations due to the size of the population and the limited genotyping approach which was not genome-wide. The combination of the 13 genetic and clinical parameters improves the current methodology. Further investigation using a genome-wide approach on a larger cohort would be necessary to capture additional weaker effects.

We identified two polymorphisms that had not previously been associated with the development of VTE. We demonstrated that they are key in the development of VTE in CC users and future studies will address their role in the general population. The first one (rs1799853) is an established genetic markers in the field of pharmacogenetics also called *2. It affects the activity of the enzyme CYP2C9 encoded by the corresponding gene. The cytochrome CYP2C9 is involved in the metabolism of ethynyl estradiol present in most of the combined pills [21]. We hypothesize that a decreased activity of the metabolism would lead to an increased systemic level of ethynyl estradiol and therefore to an increased risk of coagulation. Interestingly, the impact of this SNP on VTE in our data seems to be stronger than that of several previously described markers.

The second novel polymorphism (rs4379368) is present in the gene coding for another enzyme SUGCT. This transferase has been previously associated with migraine susceptibility using genome-wide association study [22]. It is well established that migraine is a risk factor for arterial diseases [23] and more recently migraine has also been associated with the development of VTE [24]. The combination of migraine and hormone treatment increases further the risk of cardiovascular diseases. It remains to determine, however, whether migraine as a risk factor would improve the performance of our combined model because the information was not available in our population.

The combination of the nine SNPs identified here as well as the identification of two SNPs newly associated with VTE is specific to women who use CC due to the study design. Hence further studies will be performed to confirm that these two novel SNPs and their combination would also associate with VTE in the general population.

Our model selection has three key aspects: (1) Excluding individuals with missing values would have drastically reduced the available sample size. Hence, we chose to perform 10,000 random imputations of the missing data and averaged results over the various randomly filled data sets. One could have envisioned more sophisticated data imputation, but multivariate linear imputation of the missing data would not have improved the multivariate predictive model performance. (2) To perform out-of-sample evaluation we used a cross-validation framework, where for each of the 10,000 sets we split the data into two equally-sized groups and used one group (‘training set’) to estimate the coefficients and the other group (‘test set’) to provide predictions for the AUC. We could have reported the results from a single data split, however that would not have used optimally the available data and would be prone to random fluctuations depending on the split. (3) The individual p-values of the selected 13 variables survive 5% FDR control (given the total number of tested variables); hence less than one of them is expected to be a false positive finding. Our cross-validation framework (with zeroing out the coefficients of unselected variables) was designed to protect our coefficient estimates from winners curse. Further work will establish meaningful clinical thresholds in order to translate this model into a clinical test.

In conclusion, we identified new genetic markers for VTE development among CC users and determined a new and robust combination of clinical and genetic parameters to predict VTE risk in CC users. Although further validation in independent populations should be envisaged, this combination outperforms all previously published genetic risk score in our cross-validation setting.

## Supporting information

S1 Table Polymorphism frequencies. MAF stands for Minor Allele Frequency. MAF in the 1000 genomes project is indicated as MAF 1000K. MAF in the studied population with missing values is indicated as MAF controls. Fisher test was used to estimate the deviation between both frequencies. Two SNPs present significant differences between both frequencies, they are indicated in bold.
(DOCX)

S2 Table Meta-analysis of the literature. The coefficients for each indicated variable are coming from a meta-analysis of the literature.
(DOCX)
