# Supplementary material for: Risk prediction of developing venous thrombosis in combined oral contraceptive users
Source: PLoS One. 2017 Jul 27;12(7):e0182041. doi: 10.1371/journal.pone.0182041 (PMC5531518; doi:10.1371/journal.pone.0182041)
Supplement: S1 Table — MAF stands for Minor Allele Frequency. MAF in the 1000 genomes project is indicated as MAF 1000K. MAF in the studied population with missing values is indicated as MAF controls. Fisher test was used to estimate the deviation between both frequencies. Two SNPs present significant differences between both frequencies, they are indicated in bold. (DOCX) [file pone.0182041.s001.docx]

**Supplementary information for McDaid et al.**

**S1 Table: Polymorphism frequencies**

MAF stands for Minor Allele Frequency. MAF in the 1000 genomes project is indicated as MAF 1000K. MAF in the studied population with missing values is indicated as MAF controls. Fisher test was used to estimate the deviation between both frequencies. Two SNPs present significant differences between both frequencies, they are indicated in bold.

| **SNP Accession number** | **MAF 1000K** | **MAF controls** | **p-values** |
| --- | --- | --- | --- |
| rs10029715 | 16.8 | 13.6 | 0.03 |
| rs10133762 | 47.6 | 48.5 | 0.65 |
| rs1039084 | 47.7 | 45.4 | 0.27 |
| rs1053878 | 9.9 | 7.5 | 0.03 |
| rs1063856 | 36.7 | 36.2 | 0.83 |
| rs11210892 | 36.2 | 36.6 | 0.83 |
| rs13146272 | 35.5 | 34.7 | 0.67 |
| **rs1593812** | **11.4** | **16.6** | **3.3E-04** |
| rs1613662 | 14.5 | 14.5 | 1.00 |
| rs169713 | 24.5 | 24.2 | 0.92 |
| rs169715 | 5 | 6.5 | 0.12 |
| rs1799853 | 12.4 | 11.8 | 0.66 |
| rs1799963 | 0.8 | 1.1 | 0.71 |
| rs1800595 | 6.2 | 6.4 | 0.87 |
| rs1800790 | 21.6 | 22.5 | 0.62 |
| rs1801131 | 31.3 | 29.9 | 0.48 |
| rs1801133 | 36.5 | 41.2 | 0.02 |
| rs1884841 | 44.6 | 45.9 | 0.57 |
| rs2036914 | 47.1 | 46.4 | 0.74 |
| rs2066865 | 22.1 | 24.3 | 0.21 |
| rs2227589 | 9.5 | 10.8 | 0.35 |
| rs2228220 | 11.6 | 10.4 | 0.36 |
| rs2288904 | 23.6 | 21.7 | 0.28 |
| rs2289252 | 38.9 | 41.9 | 0.14 |
| rs3136516 | 47.7 | 49 | 0.54 |
| rs3813948 | 7.1 | 9.3 | 0.05 |
| **rs429358** | **15.5** | **10.3** | **3.4E-04** |
| rs4379368 | 9.4 | 7.7 | 0.16 |
| rs4524 | 25.3 | 24.3 | 0.57 |
| rs4680 | 50 | 45.9 | 0.05 |
| rs4981021 | 26.5 | 27.2 | 0.75 |
| rs5742904 | NA | 0.1 | NA |
| rs5918 | 13.2 | 16.3 | 0.04 |
| rs5985 | 24.2 | 23.9 | 0.88 |
| rs6025 | 1.2 | 1.6 | 0.51 |
| rs6120849 | 23.2 | 23.2 | 1.00 |
| rs670659 | 38.6 | 34.8 | 0.06 |
| rs7082872 | 25 | 22.4 | 0.12 |
| rs710446 | 41.7 | 42.6 | 0.68 |
| rs8176719 | 39.5 | 40.9 | 0.48 |
| rs8176747 | 8.4 | 8.5 | 1.00 |
| rs8176750 | NA | 7 | NA |
| rs867186 | 8.7 | 10.8 | 0.10 |
| rs9380643 | 26.3 | 27.4 | 0.58 |
| rs9390459 | 46.1 | 44.3 | 0.39 |
| rs9574 | 48.3 | 48.9 | 0.81 |
