# Supplementary material for: Risk prediction of developing venous thrombosis in combined oral contraceptive users
Source: PLoS One. 2017 Jul 27;12(7):e0182041. doi: 10.1371/journal.pone.0182041 (PMC5531518; doi:10.1371/journal.pone.0182041)
Supplement: S2 Table — The coefficients for each indicated variable are coming from a meta-analysis of the literature. (DOCX) [file pone.0182041.s002.docx]

**Supplementary information for McDaid et al.**

**S2 Table: Meta-analysis of the literature**

The coefficients for each indicated variable are coming from a meta-analysis of the literature (1-23)

.

| **Variable** | **Coefficient** |
| --- | --- |
| Smoking status | 1.6 |
| Familial history | 2 |
| BMI ≥25 and < 30 | 1.43 |
| BMI ≥30 | 2.31 |
| FV-Leiden heterozygous | 4.11 |
| homozygous | 11.15 |
| Prothrombin F2 heterozygous | 3.5 |
| homozygous | 8.4 |

Supplementary references for S2 Table:

1. Aznar J, Vaya A, Estelles A, Mira Y, Segui R, Villa P, et al. Risk of venous thrombosis in carriers of the prothrombin G20210A variant and factor V Leiden and their interaction with oral contraceptives. Haematologica. 2000;85(12):1271-6.

2. Bezemer ID, Bare LA, Doggen CJ, Arellano AR, Tong C, Rowland CM, et al. Gene variants associated with deep vein thrombosis. JAMA. 2008;299(11):1306-14.

3. Borch KH, Braekkan SK, Mathiesen EB, Njolstad I, Wilsgaard T, Stormer J, et al. Anthropometric measures of obesity and risk of venous thromboembolism: the Tromso study. Arterioscler Thromb Vasc Biol. 2010;30(1):121-7.

4. Cattaneo M, Chantarangkul V, Taioli E, Santos JH, Tagliabue L. The G20210A mutation of the prothrombin gene in patients with previous first episodes of deep-vein thrombosis: prevalence and association with factor V G1691A, methylenetetrahydrofolate reductase C677T and plasma prothrombin levels. Thromb Res. 1999;93(1):1-8.

5. Catto AJ, Kohler HP, Coore J, Mansfield MW, Stickland MH, Grant PJ. Association of a common polymorphism in the factor XIII gene with venous thrombosis. Blood. 1999;93(3):906-8.

6. Delluc A, Le Moigne E, Tromeur C, Noel-Savina E, Couturaud F, Mottier D, et al. Site of venous thromboembolism and prothrombotic mutations according to body mass index. Results from the EDITH study. Br J Haematol. 2011;154(4):486-91.

7. Di Nisio M, Di Iorio A, Porreca E, Abate M, Ferrante N, Bandinelli S, et al. Obesity, poor muscle strength, and venous thromboembolism in older persons: the InCHIANTI study. J Gerontol A Biol Sci Med Sci. 2011;66(3):320-5.

8. Emmerich J, Rosendaal FR, Cattaneo M, Margaglione M, De Stefano V, Cumming T, et al. Combined effect of factor V Leiden and prothrombin 20210A on the risk of venous thromboembolism--pooled analysis of 8 case-control studies including 2310 cases and 3204 controls. Study Group for Pooled-Analysis in Venous Thromboembolism. Thromb Haemost. 2001;86(3):809-16.

9. Gohil R, Peck G, Sharma P. The genetics of venous thromboembolism. A meta-analysis involving approximately 120,000 cases and 180,000 controls. Thromb Haemost. 2009;102(2):360-70.

10. Holst AG, Jensen G, Prescott E. Risk factors for venous thromboembolism: results from the Copenhagen City Heart Study. Circulation. 2010;121(17):1896-903.

11. Juul K, Tybjaerg-Hansen A, Schnohr P, Nordestgaard BG. Factor V Leiden and the risk for venous thromboembolism in the adult Danish population. Ann Intern Med. 2004;140(5):330-7.

12. Parkin L, Sweetland S, Balkwill A, Green J, Reeves G, Beral V, et al. Body mass index, surgery, and risk of venous thromboembolism in middle-aged women: a cohort study. Circulation. 2012;125(15):1897-904.

13. Pomp ER, le Cessie S, Rosendaal FR, Doggen CJ. Risk of venous thrombosis: obesity and its joint effect with oral contraceptive use and prothrombotic mutations. Br J Haematol. 2007;139(2):289-96.

14. Poort SR, Rosendaal FR, Reitsma PH, Bertina RM. A common genetic variation in the 3'-untranslated region of the prothrombin gene is associated with elevated plasma prothrombin levels and an increase in venous thrombosis. Blood. 1996;88(10):3698-703.

15. Renner W, Koppel H, Hoffmann C, Schallmoser K, Stanger O, Toplak H, et al. Prothrombin G20210A, factor V Leiden, and factor XIII Val34Leu: common mutations of blood coagulation factors and deep vein thrombosis in Austria. Thromb Res. 2000;99(1):35-9.

16. Rosendaal FR, Koster T, Vandenbroucke JP, Reitsma PH. High risk of thrombosis in patients homozygous for factor V Leiden (activated protein C resistance). Blood. 1995;85(6):1504-8.

17. Rosendaal FR, Vessey M, Rumley A, Daly E, Woodward M, Helmerhorst FM, et al. Hormonal replacement therapy, prothrombotic mutations and the risk of venous thrombosis. Br J Haematol. 2002;116(4):851-4.

18. Severinsen MT, Kristensen SR, Johnsen SP, Dethlefsen C, Tjonneland A, Overvad K. Smoking and venous thromboembolism: a Danish follow-up study. J Thromb Haemost. 2009;7(8):1297-303.

19. Severinsen MT, Overvad K, Johnsen SP, Dethlefsen C, Madsen PH, Tjonneland A, et al. Genetic susceptibility, smoking, obesity and risk of venous thromboembolism. Br J Haematol. 2010;149(2):273-9.

20. Sode BF, Allin KH, Dahl M, Gyntelberg F, Nordestgaard BG. Risk of venous thromboembolism and myocardial infarction associated with factor V Leiden and prothrombin mutations and blood type. CMAJ. 2013;185(5):E229-37.

21. Tregouet DA, Heath S, Saut N, Biron-Andreani C, Schved JF, Pernod G, et al. Common susceptibility alleles are unlikely to contribute as strongly as the FV and ABO loci to VTE risk: results from a GWAS approach. Blood. 2009;113(21):5298-303.

22. Tsai AW, Cushman M, Rosamond WD, Heckbert SR, Polak JF, Folsom AR. Cardiovascular risk factors and venous thromboembolism incidence: the longitudinal investigation of thromboembolism etiology. Arch Intern Med. 2002;162(10):1182-9.

23. Wattanakit K, Lutsey PL, Bell EJ, Gornik H, Cushman M, Heckbert SR, et al. Association between cardiovascular disease risk factors and occurrence of venous thromboembolism. A time-dependent analysis. Thromb Haemost. 2012;108(3):508-15.
